# Novel likely pathogenic variants in *TMEM126A* identified in non-syndromic autosomal recessive optic atrophy: two case reports

**DOI:** 10.1186/s12881-019-0795-x

**Published:** 2019-04-08

**Authors:** Katja Kloth, Matthis Synofzik, Christoph Kernstock, Simone Schimpf-Linzenbold, Frank Schuettauf, Axel Neu, Bernd Wissinger, Nicole Weisschuh

**Affiliations:** 1 Institute of Human Genetics, University Medical Center Hamburg-Eppendorf, Hamburg, Germany; 2 Department of Neurodegenerative Diseases, Hertie Institute for Clinical Brain Research, University of Tübingen, Tübingen, Germany; 3 German Center for Neurodegenerative Diseases (DZNE), Tübingen, Germany; 4 Center for Ophthalmology, Institute for Ophthalmic Research, University of Tübingen, Tübingen, Germany; 5 CeGaT GmbH and Praxis für Humangenetik Tübingen, Tübingen, Germany; 6 Department of Ophthalmology, University Medical Center Hamburg-Eppendorf, Hamburg, Germany; 7 Department of Pediatrics, University Medical Center Hamburg-Eppendorf, Hamburg, Germany

**Keywords:** Optic atrophy, Autosomal recessive, TMEM126A

## Abstract

**Background:**

Reports on autosomal recessive optic atrophy (arOA) are sparse and so far, only one gene has been specifically associated with non-syndromic arOA, namely *TMEM126A*. To date, all reports of pathogenic *TMEM126A* variants are from affected individuals of Maghrebian origin, who all carry an identical nonsense variant. Here we report two novel variants in the *TMEM126A* gene from non-Maghreb individuals, both found in affected individuals with an arOA phenotype.

**Case presentation:**

We report three affected individuals from two families. The proband of family A, a 24-year-old Turkish woman, was diagnosed with visual loss in early childhood but a diagnosis of optic atrophy was only made at 14 years. A diagnostic gene panel revealed a splice donor variant (c.86 + 2 T > C) in homozygous state in the *TMEM126A* gene. Analysis of this variant based on RNA from whole blood revealed a single aberrant transcript lacking exon 2, presumably representing a functional null allele. Two siblings from family B, a 16-year old Iraqi girl and her 14-year old brother, were diagnosed with optic atrophy in early childhood. A missense variant p.(S36 L) in the *TMEM126A* gene was identified in homozygous state in a gene panel-based diagnostic setting in both siblings. This missense variant is ultra rare in the general population, affects a highly evolutionarily conserved amino acid and segregates with the disease within the family. The three probands reported in this study had a relatively mild clinical course without any evidence of a syndromic (e.g. neurological) comorbidity, which is in line with previous studies.

**Conclusions:**

We provide additional evidence for the implication of biallelic pathogenic *TMEM126A* variants in arOA. Our findings extend both the mutational spectrum and geographic presence of *TMEM126A* in arOA. Screening of the entire gene should be considered in affected individuals presenting with features resembling arOA and also from non-Maghrebian descent.

## Background

Inherited optic neuropathies are rare ocular diseases that are characterized by early-onset, slowly progressive and bilateral vision loss, central scotoma and color vision disturbances due to dysfunction and degeneration of retinal ganglion cells and the optic nerve [1]. Most of the published genes and pathogenic variants have been associated with autosomal-dominant optic atrophy (adOA, MIM 165500) or maternally inherited Leber hereditary optic neuropathy (LHON, MIM 535000). To this day, only one gene has been specifically associated with non-syndromic autosomal-recessive atrophy (arOA), namely *TMEM126A* [2]. This gene has been mapped to chromosome 11q14.1, covers a genomic region of 8.5 kB (GenBank NM_032273) and is composed of five exons. So far, all affected individuals reported in the literature originate from the same region, the Maghreb, and are homozygous for a recurrent nonsense variant (p.R55*) in the *TMEM126A* gene [2–4].

There is an intimate relationship between optic atrophy disease genes and mitochondrial function, since virtually all publicly known disease genes encode proteins that are localized in mitochondria and/or play a role in mitochondrial homeostasis. The exact function of the TMEM126A protein remains unknown. However, extensive studies performed by Hanein and coworkers have shown that it is a protein of the mitochondrial inner membrane with its N- and C-termini facing the mitochondrial matrix [5]. While affected individuals with pathogenic variants in mitochondrial DNA often clinically present with a multi-systemic disorder, pathogenic variants in nuclear-encoded mitochondrial proteins may be associated with single-system dysfunctions such as adOA caused by pathogenic variants in *OPA1*, a nuclear encoded mitochondrial large GTPase of the dynamin family that plays a central role in mitochondrial dynamics and cristae junction maintenance [6–8]. Progressive visual loss is the most common symptom in these affected individuals resulting from a dysfunction and eventually loss of retinal ganglion cells and their axons. Recent findings, however, showed that the disease spectrum due to pathogenic *OPA1* variants is much broader and includes a variety of syndromic diseases such as adOAplus featuring hearing loss, cerebellar ataxia, ptosis, and peripheral neuropathy or a form of Behr syndrome with additional neuromuscular deficits, partly even with biallelic inheritance [9, 10]. While individuals with pathogenic variants in *TMEM126A* have been initially reported to be affected with non-syndromic optic atrophy [2], subsequent studies have shown that some affected individuals also presented with mild extra-ocular symptoms such as auditory neuropathy [3] or sensory-motor axonal neuropathy [4].

In this study, we report three probands from two families harboring two novel putative pathogenic variants in *TMEM126A.* In-depth neurological examination revealed no syndromic involvement in these affected individuals. Our results emphasize the need for sequencing the entire *TMEM126A* gene in affected individuals with arOA instead of focusing on the recurrent (p.R55*) variant, and testing also affected individuals outside from Maghreb. Moreover, the identification of a first missense variant may provide a hint on protein domains crucial for the mitochondrial function of TMEM126A.

## Case presentation

### Affected individuals

Family A was recruited and clinically examined at the Centre for Ophthalmology and at the Department of Neurodegenerative Diseases, University of Tübingen, Germany. Ethical approval (dated October 23, 2017; Project number 637/2017BO1) and informed consent for all individuals was obtained. DNA from whole blood was extracted from all family members using standard protocols. Total RNA was isolated from whole blood drawn in PAXgene tubes using the PAXgene blood RNA Kit (Qiagen, Hilden, Germany).

Family B was recruited and clinically examined at the Center for Ophthalmology and at the Institute of Human Genetics, University Medical Center Hamburg-Eppendorf, Hamburg, Germany. Ethical approval (dated March 1, 2016; Project number PV3802) and informed consent for all individuals including research purposes was obtained. DNA from whole blood was extracted from all family members using standard protocols.

### Clinical investigation

Ophthalmic examination of the affected probands of family A (II:1) and family B (II:1 and II:2) included fundus photography, best corrected visual acuity (BCVA, Snellen), measurement of intraocular pressure, perimetry and spectral domain optical coherence tomography (SD-OCT; Heidelberg Engineering GmbH, Heidelberg, Germany). Details on methodology have already been described [10, 11]. Neurological examination of the affected probands of family A (II:1) and family B (II:1, II:2) included assessment of cognitive function, primary sensory modalities, reflexes, coordination and gait. Nerve conduction studies were performed in subject A (II:1). Magnetic resonance imaging (MRI) of the head was also performed in proband II:1 of family A and proband II:1 of family B.

### Panel sequencing and data filtering

Sequencing libraries were prepared starting from DNA using the SureSelectXT workflow (Agilent, Santa Clara, CA) and a custom-design enrichment kit (CeGaT EYE, version 8) for the index patients. Library preparation and capture was performed according to the manufacturer’s instructions and paired-end sequencing was performed on a HiSeq4000 instrument (Illumina, San Diego, CA) (for family A) or on a NovaSeq6000 instrument (Illumina, San Diego, CA) (for family B) with 2 × 100 base pairs (bp) read length, yielding 5 Gbp and 5.3 Gbp of data, respectively. After demultiplexing (Illumina bcl2fastq 2.19), adapters were trimmed with Skewer version 0.1.116 [12]. Trimmed raw reads were aligned to the human genome (hg19) with the Burrows-Wheeler Aligner (BWA-mem version 0.7.2) [13]. Reads likely arising from PCR duplication as well as reads that aligned at more than one locus were discarded (CeGaT internal software). Average coverage on target was 522x (99.2% >10x, 99.53% >30x) for family A and 1145x (99.7% >10x, 99.7% >30x) for family B. Sequence variants were called (VarScan 2.4.2, CeGaT extended version) with a minimum variant allele frequency of 5%. Calls resulting from technical artefacts were removed (CeGaT internal software). Resulting variants were annotated with population frequencies from dbSNP (release 149 and 150) [14], ExAC version 0.3.1 [15] (for family A), gnomAD version 2.0.1 [15] (for family B) and an internal database (CeGaT), with functional predictions from dbNSFP (3.0c and 3.4c) [16], with publications from HGMD (16.4 and 17.2) [17], and with transcript information from Ensembl [18], RefSeq [19], and CCDS [20]. Variants were filtered to remove frequent variants (minor allele frequency 1.5%). All variants were manually assessed before inclusion in the final report. Analysis of family members (segregation analysis) was performed by standard Sanger sequencing.

### cDNA analysis

Total RNA isolated from whole blood was reverse transcribed using the Transcriptor First Strand cDNA Synthesis Kit (Roche, Mannheim, Germany) according to the manufacturer’s recommendations. The cDNA was PCR amplified using a forward primer located in exon 1 of *TMEM126A* (GTGGCTGAGGAAGGAGGAG) and a reverse primer located in exon 4 (CAGTCCACTCCGTGTTATGG). Sequence analysis of RT-PCR products was performed as described previously [21].

### Clinical findings

**Fig. 1:**
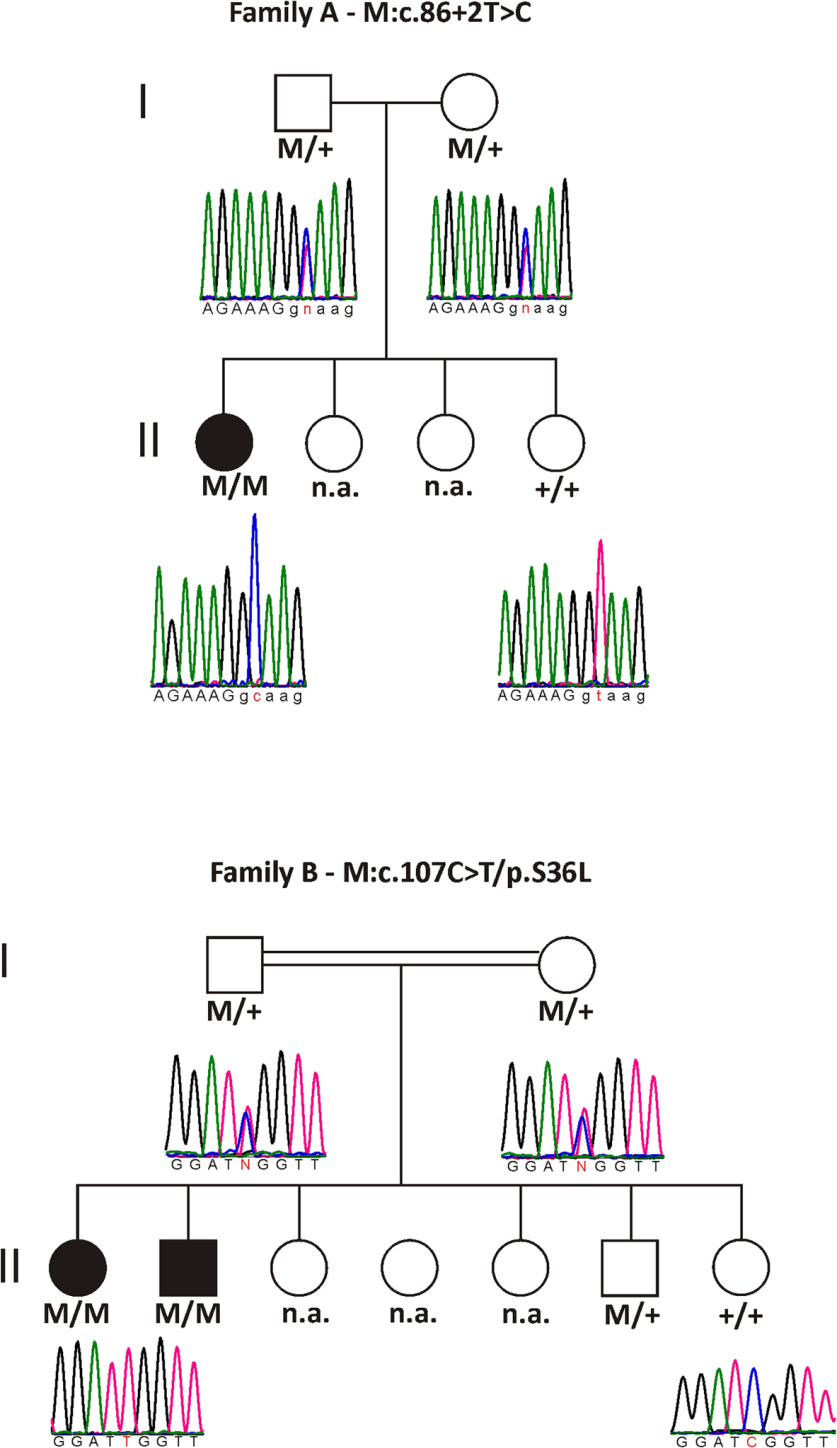
Pedigrees of families A and B. Genotypes and sequence profiles are given below each available family member. Exonic sequences are given in capital letters and intronic sequences in small letters, respectively. The nucleotides that are changed are highlighted in red. M, mutant allele; +, wildtype allele; n.a., not analyzed

**Fig. 2:**
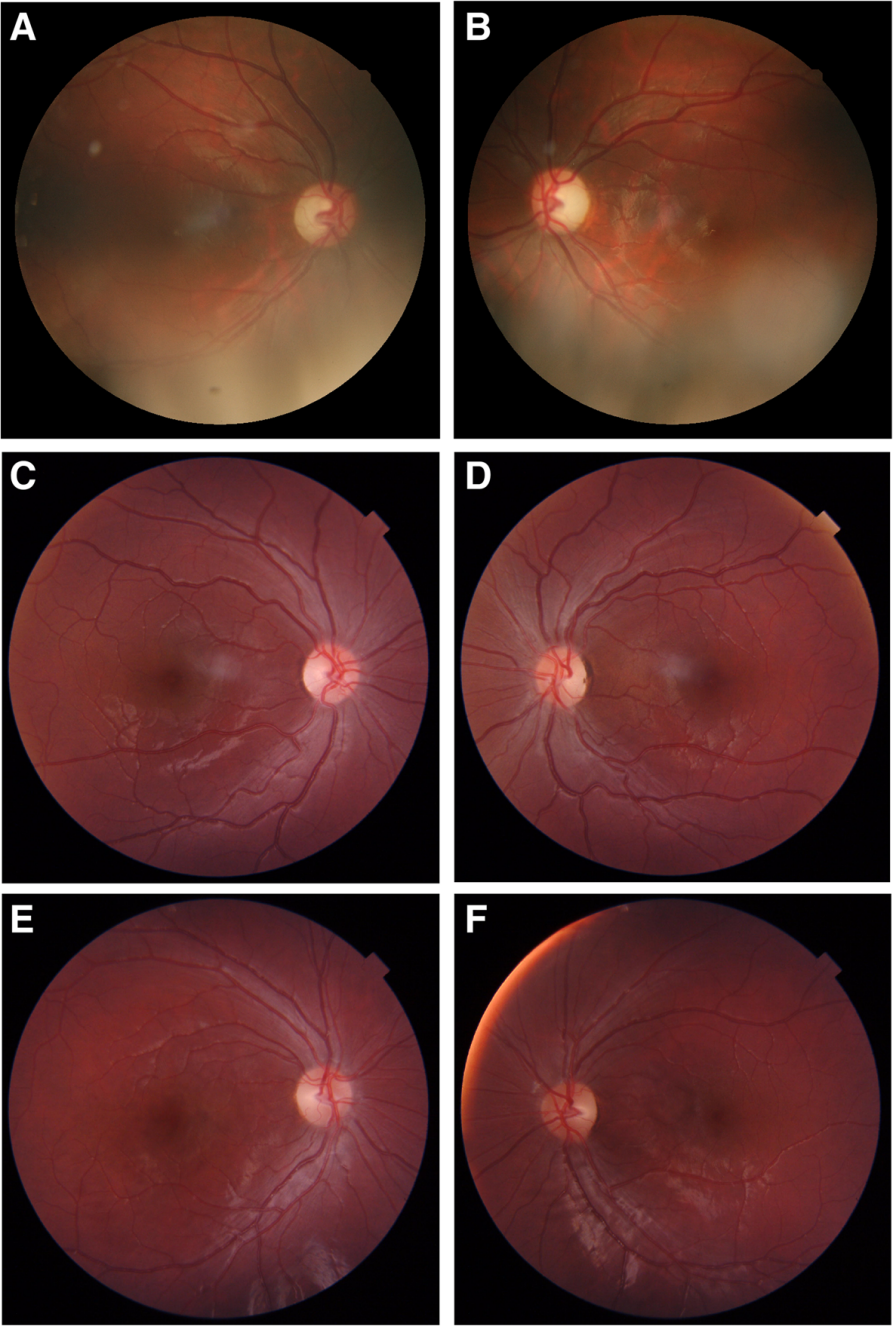
Eye fundi. Temporal pallor of discs is seen in the right eye (**a**) and the left eye (**b**) of the affected proband in family A (II:1) as well as in the affected sister (**c** + **d**; II:1) and her brother (**e** + **f**; II:2) of family B

**Fig. 3:**
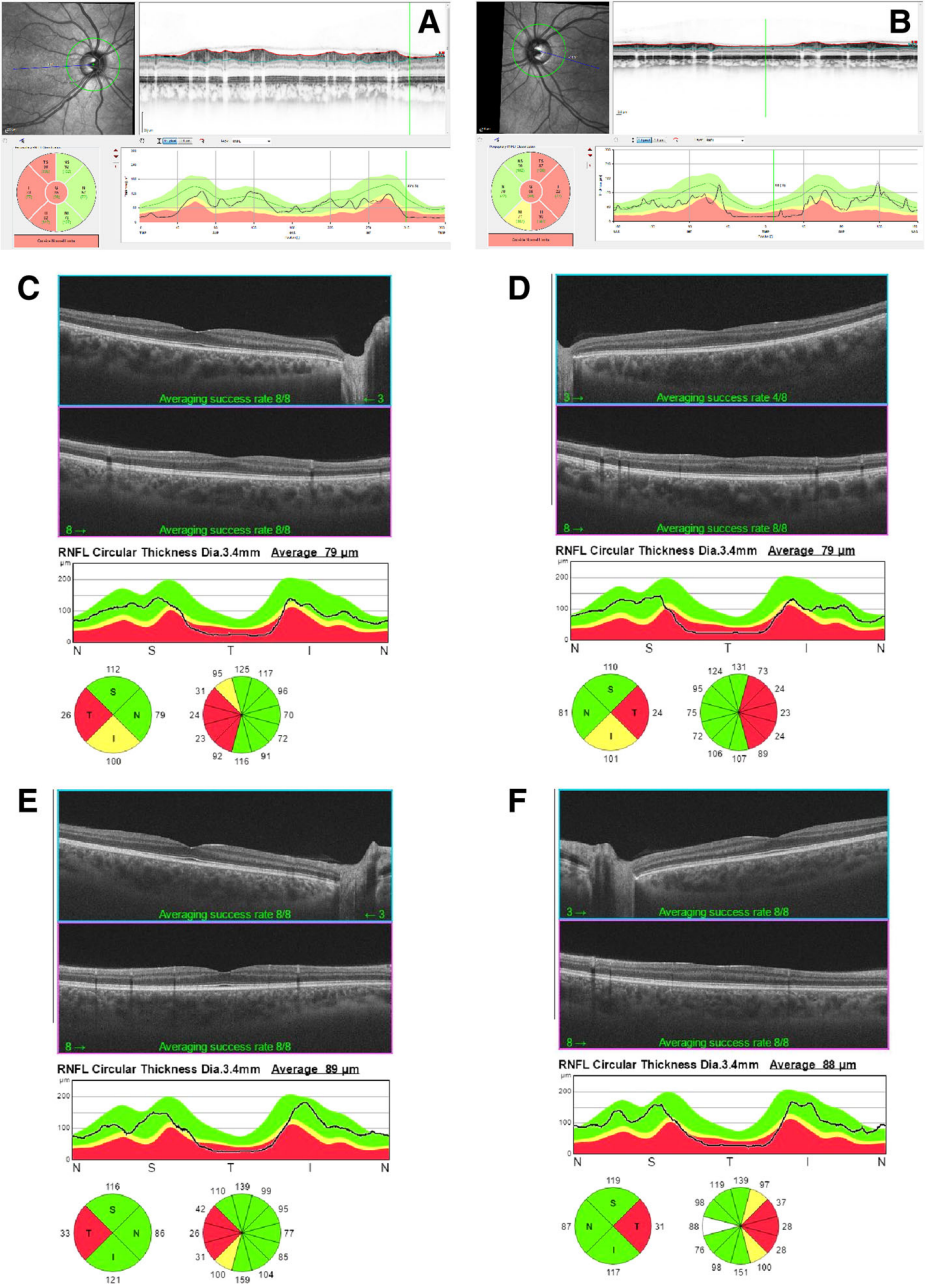
Optical coherence tomography (OCT) of the retinal nerve fiber layers (RNFLs). (**a**-**b**) The right eye (**a**) and left eye (**b**) OCT of the affected proband in family A (II:1) show temporal thinning of RNFLs. This can also be seen in the affected sister (**c**-**d**; II:1) and her brother (**e**-**f**; II:2) of family B

**Fig. 4:**
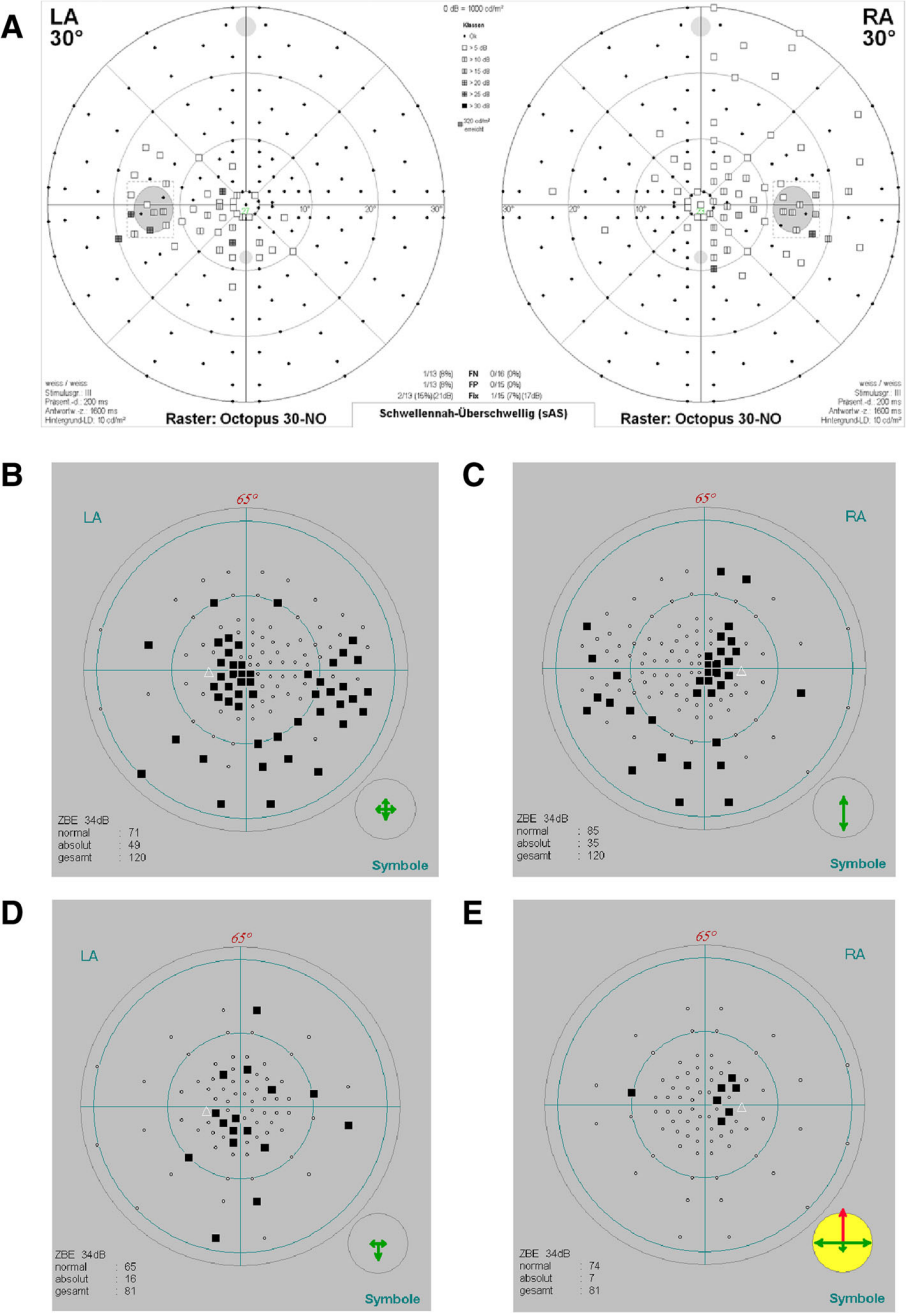
Perimetry. **a** The right and left eye visual fields are represented for the affected proband in family A (II:1) and exhibit central scotomas. Scotomas can also be seen in the affected sister (**b**-**c**; II:1) and her brother (**d**-**e**; II:2) of family B, with the sister showing a more pronounced visual field loss

The affected proband from family A is a 24-year-old Turkish woman, born to unaffected parents (Fig. 1, II:1 in the pedigree). Consanguinity was not reported. Family history revealed three healthy sisters. At age 6, the proband noticed a decrease in visual acuity but a diagnosis of optic atrophy was only made at 14 years of age. Fundus examination at 24 years of age revealed pale discs (see Fig. 2a + b). Visual acuity was reduced to 20/100 (OD) and 20/200 (OS). Optical coherence tomography showed a markedly reduced peripapillary retinal nerve fiber layer in the temporal sector on both eyes (see Fig. 3a + b). Perimetry showed centrocecal relative scotomas consistent with papillary atrophy (see Fig. 4a).

Neurological in-depth examination by a neurological expert with long-standing experience in examining neurogenetic patients (M.S.) revealed no clinical signs of damage to any neurological system, in particular not of the pyramidal, basal ganglia, cerebellar, spinal or peripheral nerve system. Nerve conduction studies showed normal results for sural and tibial nerves. Also her cognitive abilities were within the full normal range, with the proband finishing regular school and working full-time on the regular job market as a cashier in a grocery store.

The affected probands from family B are a 16-year old Iraqi girl and her 14-year old brother, born to unaffected consanguineous parents (Fig. 1, Family B, I:1 and I:2). Family history reported four healthy sisters (II:3–5; II:7) and one healthy brother (II:6). A grandfather on the mother’s side was reported to suffer from blindness at the age of 20 years. The girl was initially diagnosed with poor vision and achromatopsia in early childhood. The clinical diagnosis was revised to optic atrophy when visual evoked potentials showed reduced potentials and delayed latency periods at the age of 14. Fundus examination at age 15 revealed temporal pallor of the optic discs and the macular reflex was diminished (see Fig. 2c + d). Visual acuity at this point was reduced to 20/100 (proximity) and 50/100 (distance). Peripapillary retinal nerve fiber layer showed temporal thinning similar to proband A (see Fig. 3c + d). Perimetry showed central and paracentral defects. Additionally, arcuated defects were recorded within the temporal visual fields (see Fig. 4b + c) that could not be attributed to the physiognomy of the proband. The boy was diagnosed during infancy with reduced visual acuity in one eye and was reported to suffer from nyctalopia. Ophthalmological examination revealed slight temporal pallor of the optic discs and the macular reflex was diminished (see Fig. 2e + f). Visual acuity at this point was 25/100 (proximity) and 10/100 (distance). Peripapillary retinal nerve fiber layer showed temporal thinning (see Fig. 3e + f). Perimetry showed paracentral defects (see Fig. 4d + e).

Neurological in-depth examination of both children by a trained neuropediatrician and a skilled clinical geneticist revealed no signs of neurological disease or dysfunction, in particular not of the pyramidal, basal ganglia, cerebellar, spinal or peripheral nerve system. Their cognitive abilities were within the full normal range, with both affected individuals effectively attending regular schooling.

### Genetic findings

DNA of both index patients was analyzed using a panel-based sequencing approach in a diagnostic setting. The gene panel targets 16 genes that are associated with optic atrophy, namely *OPA1*, *OPA3*, *TMEM126A*, *WFS1*, *MFN2*, *TIMM8A*, *SPG7*, *NR2F1*, *ACO2*, *RTN4IP1*, *AFG3L2*, *C12ORF65*, *SLC25A46*, *CISD2*, *DNM1L*, and *YME1L1*. No rare and potentially disease-causing exonic variants explaining the disease phenotype were found except for a canonical splice donor site variant in *TMEM126A* in family A and a missense variant in the same gene in family B. Both variants segregate with the disease in the families as can be seen in the pedigrees shown in Fig. 1.

**Fig. 5:**
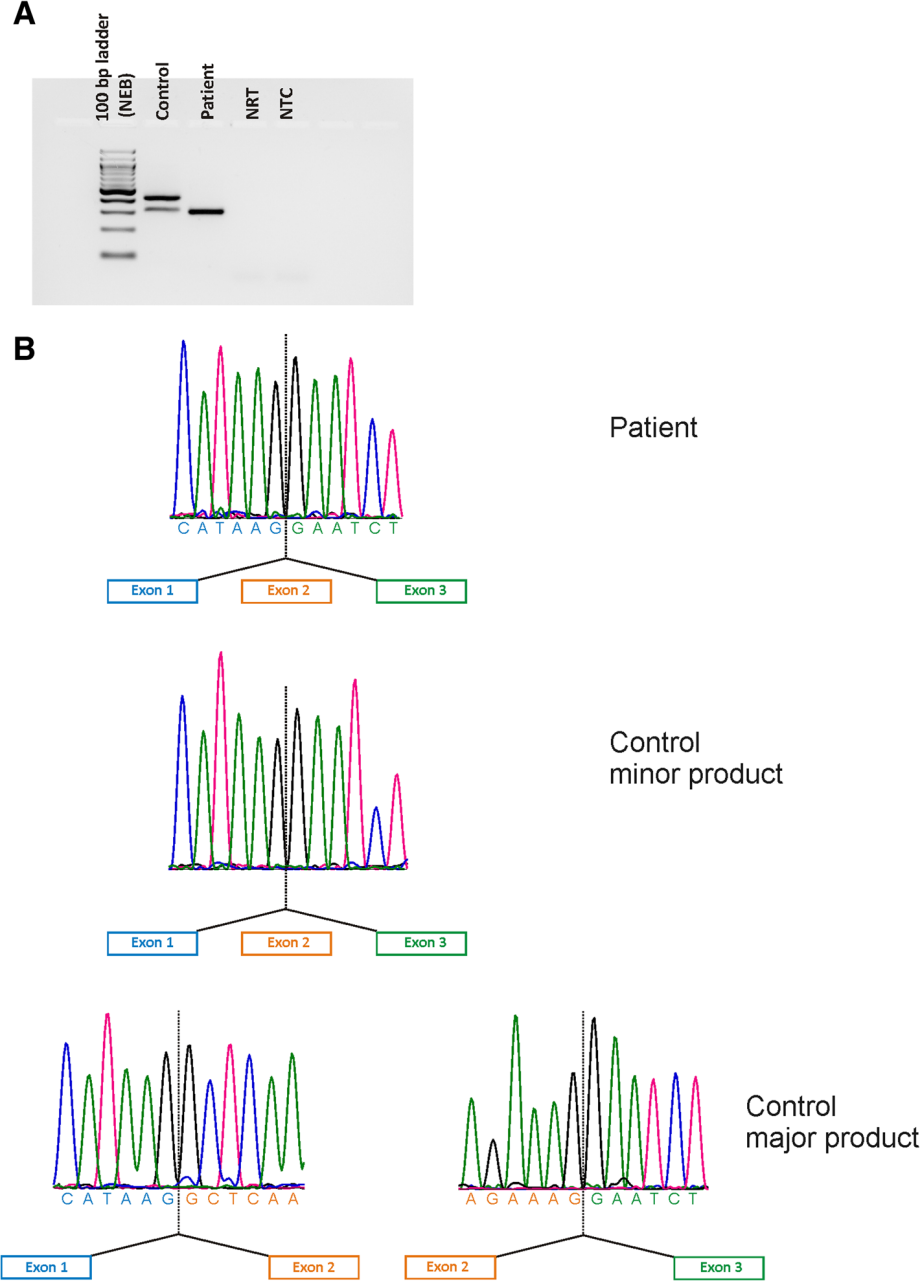
cDNA analysis of the c.86 + 2 T > C variant. **a** RT-PCR of an unrelated control subject (lane 2) using a forward primer located in exon 1 and a reverse primer in exon 4 revealed an abundant product of 382 bp corresponding to the major isoform of *TMEM126A*. In addition, a weak product could be identified, which stems from a minor isoform that lacks exon 2. The RT-PCR of the affected proband in family A (II:1) shows that only the minor isoform is present (lane 3). **b** Sequencing shows skipping of exon 2 in the single RT-PCR product of the affected proband (upper lane) and for the minor RT-PCR product of an unrelated control subject (middle lane) while correct splicing is observed for the abundant RT-PCR product in an unrelated control subject (lower lane). RT-PCR: reverse transcription polymerase chain reaction; NRT, no reverse transcriptase control; NTC, non template control

The splice donor variant c.86 + 2 T > C found in family A, which is not present in large population databases (dbSNP, gnomAD browser), directly changes the invariant 5′ splice core GT sequence of intron 2, ablating the site, with anticipated effects on mRNA splicing. Subsequent cDNA analysis based on RNA isolated from whole blood revealed a single transcript with complete skipping of exon 2 (see Fig. 5). As exon 2 contains the translation start site, the variant c.86 + 2 T > C presumably results in the failure of translation from its natural start site. There is a shorter isoform of TMEM126A, using an ATG in exon 3, which codes for a protein of 125 aa (Ensembl Transcript ENST00000528105.5). This isoform is observed in normal controls, though the larger isoform (Ensembl Transcript ENST00000304511.6) with the ATG in exon 2 is much more abundant (see Fig. 5a). This larger isoform is completely lacking in the proband homozygous for the c.86 + 2 T > C variant.

The p.(S36 L) variant identified in family B is extremely rare in the general population with a minor allele frequency of 8.122e-6 (sourced from gnomAD). Two different online tools were used to assess the potential pathogenicity of the p.(S36 L) variant, namely the UMD predictor (version March 2018) [22] and the Combined Annotation Dependent Depletion tool (version 1.2) [23]. Within the UMD predictor, scores range from 0 to 100, with scores > 74 indicating pathogenicity of a variant. The Combined Annotation Dependent Depletion tool uses a different scoring system with scores > 20 predicting pathogenicity of a variant. The analysis of the p.(S36 L) variant using both tools resulted in scores of 93 and 25, respectively, thereby emphasizing the putative pathogenicity of this novel missense variant.

## Discussion and conclusions

The homozygous nonsense variant (p.R55*) in *TMEM126A* was proposed as the genetic defect underlying arOA in a large multiplex inbred Algerian family [2]. Clinical features included early-onset severe bilateral deficiency in visual acuity, optic disc pallor, and central scotoma. Subsequently, additional cases, all of Maghrebian descent, carrying the same founder mutation were reported [2–4].

In the current manuscript, we provide further evidence for the implication of biallelic pathogenic *TMEM126A* variants in arOA, as we diagnosed two additional cases with presumably pathogenic variants in this gene. The index patient of family A, of Turkish descent, carried a novel canonical splice site variant c.86 + 2 T > C, while two affected siblings from family B, of Iraqi descent, carried a novel missense variant, p.(S36 L). Affected members from both families presented with typical symptoms of optic atrophy but revealed no syndromic involvement.

TMEM126A (NP_115649) is predicted to contain four transmembrane (TM) domains with the first being formed by amino acid (aa) residues 39–57 [5]. The function of the N-terminus (aa 1–38) is completely unknown. One possibility could be that it acts as a mitochondrial targeting leader sequence. However, mitochondrial presequences are usually rich in positively charged aa residues, they have the potential to form amphiphilic alpha-helices and contain consensus motifs for cleavage sites [24], neither of which holds true for the N-terminus of TMEM126A. In fact, it has been shown that it is the second transmembrane domain (aa 66–85) that promotes localization to the mitochondria [5]. While it seems obvious that the p.(S36 L) variant does not influence the localization to the mitochondria, it may exert a pathogenic effect through another mechanism. Missense variants may not only act at the protein level but also at the nucleotide level by interfering with the correct assembly of the pre-mRNA splicing machinery. However, in silico analysis of the p.(S36 L) variant using the Human Splicing Finder version 3.0 [25] revealed no difference between mutant and reference sequence. Due to the lack of functional data, and according to the guidelines for the classification of sequence variants by the American College of Medical Genetics and Genomics and the Association for Molecular Pathology [26], the p.(S36 L) variant has to be categorized as variant of unclear significance, despite the fact that it represents an ultra-rare variant in the general population, segregates with the disease in the family and affects an evolutionary conserved amino acid residue. On the other hand, we could demonstrate that the canonical splice site variant c.86 + 2 T > C leads to the expression of only the short transcript isoform which, if translated, would lack the first seventy amino acids of the canonical protein. This would impair the formation of the second transmembrane domain and we hypothesize that this shortened protein would not be located to the mitochondria.

In conclusion, more information on the effect of TMEM126A alterations on mitochondrial function is needed to confirm the exact pathogenic mechanisms that cause arOA in the probands of this study. Taking our data and the previous publications into account, *TMEM126A* remains the most promising candidate for non-syndromic arOA and should be considered in affected individuals with a corresponding phenotype (non-syndromic optical atrophy with or without minor extra-ocular findings such as cardiomyopathy, hearing loss, MRI alterations), also in affected individuals outside Maghreb.

## Abbreviations

aa: Amino acid
adOA: Autosomal-dominant optic atrophy
arOA: Autosomal recessive optic atrophy
LHON: Leber Hereditary Optic Neuropathy
MRI: Magnetic resonance imaging
OCT: Optical coherence tomography
OPA1: Optic atrophy 1
RNFL: Retinal nerve fiber layer
RT-PCR: Reverse transcription polymerase chain reaction
TM: Transmembrane
TMEM126A: Transmembrane protein 126A
